# Comparing ambient, air-convection, and fluid-convection heating techniques in treating hypothermic burn patients, a clinical RCT

**DOI:** 10.1186/1750-1164-5-4

**Published:** 2011-07-07

**Authors:** Britt-Marie Kjellman, Mats Fredrikson, Gunilla Glad-Mattsson, Folke Sjöberg, Fredrik RM Huss

**Affiliations:** 1 The Burn unit, Dept. of Plastic Surgery, Hand Surgery and Burns, University Hospital of Linköping, Linköping, Sweden; 2 Department of Clinical and Experimental Medicine, Faculty of Health Sciences, Linköping University, Linköping, Sweden; 3 Department of Surgical Sciences, Plastic Surgery, Uppsala University, Uppsala, Sweden

## Abstract

**Background:**

Hypothermia in burns is common and increases morbidity and mortality. Several methods are available to reach and maintain normal core body temperature, but have not yet been evaluated in critical care for burned patients. Our unit's ordinary technique for controlling body temperature (Bair Hugger^®^+ radiator ceiling + bed warmer + Hotline^®^) has many drawbacks e.g.; slow and the working environment is hampered.

The aim of this study was to compare our ordinary heating technique with newly-developed methods: the Allon™2001 Thermowrap (a temperature regulating water-mattress), and Warmcloud (a temperature regulating air-mattress).

**Methods:**

Ten consecutive burned patients (> 20% total burned surface area and a core temperature < 36.0°C) were included in this prospective, randomised, comparative study. Patients were randomly exposed to 3 heating methods. Each treatment/measuring-cycle lasted for 6 hours. Each heating method was assessed for 2 hours according to a randomised timetable. Core temperature was measured using an indwelling (bladder) thermistor. Paired *t*-tests were used to assess the significance of differences between the treatments within the patients. ANOVA was used to assess the differences in temperature from the first to the last measurement among all treatments. Three-way ANOVA with the Tukey HSD *post hoc *test and a repeated measures ANOVA was used in the same manner, but included information about patients and treatment/measuring-cycles to control for potential confounding. Data are presented as mean (SD) and (range). Probabilities of less than 0.05 were accepted as significant.

**Results:**

The mean increase, 1.4 (SD 0.6°C; range 0.6-2.6°C) in core temperature/treatment/measuring-cycle highly significantly favoured the Allon™2001 Thermowrap in contrast to the conventional method 0.2 (0.6)°C (range -1.2 to 1.5°C) and the Warmcloud 0.3 (0.4)°C (range -0.4 to 0.9°C). The procedures for using the Allon™2001 Thermowrap were experienced to be more comfortable and straightforward than the conventional method or the Warmcloud.

**Conclusions:**

The Allon™2001 Thermowrap was more effective than the Warmcloud or the conventional method in controlling patients' temperatures.

## Background

Transient hypothermia (i.e. low body core temperature) is common in burns. All patients risk a decrease in body temperature between the scene of trauma and admission to the burn unit. At the burn unit repeated procedures under anaesthesia mean that the risk of hypothermia is not reduced for a long part of the patient's stay. Despite this risk, little progress has been made to resolve the issue.

In our burn unit the typical approach to combat hypothermia would be increased ambient room temperature, resuscitation with warm fluids, warm blankets, radiators in the ceilings, and hot air. These techniques are often easily available and technically less demanding but often not effective enough and slow working as well as influencing the staff's work environment.

For hypothermic trauma patients a number of rewarming modalities have been described and can be divided in three main strategies; *passive rewarming *(optimizing the environment thus allowing endogenous heat production), active external rewarming (adding heat to body surfaces), and active core rewarming (adding heat to internal body surfaces) [1].

Even though passive rewarming could be considered the basic first step in rewarming a cold patient it alone is seldom efficient enough in burn care and should be combined with other modalities. Active external rewarming by e.g. convective air blankets is routinely used in burn care. However, peripherally vasoconstricted patients are often less susceptible to active external rewarming and can also sustain thermal injuries by intense local heat build-up, especially in already traumatized (burned) skin. Probably the most frequently, and easiest, used active core rewarming technique in burn care is intravenous infusion of warm fluids. There are, though, more elaborate examples such as e.g. body-cavity lavage and airway rewarming available.

Any of the three rewarming strategies may be appropriate in certain settings and in situations where resource availability varies.

However, hypothermia during admission and procedures are still reported [2-7], even though numerous (new) rewarming approaches, including invasive techniques, have been described [5,8-13].

Invasive techniques using e.g. intravascular thermal regulation catheters have been proposed to be effective and reliable [13]. However, invasive techniques are not commonly available and certainly more technical challenging at least regarding burn patients. Catheters need to be introduced centrally in highly bacterial susceptible patients and its safety and efficacy is still to be investigated in burn patients.

Hypothermia is associated with several deleterious effects and increased morbidity and mortality. Complications such as myocardial ischaemia, arrhythmias, vasoconstriction, and coagulopathies are associated with hypothermia, as is impairment of wound healing, and abnormalities of the immune-, stress-, and neurological systems [8,14-18].

The patient's core temperature is defined as the temperature in the central circulation, and is regulated by the hypothalamus. The core temperature in a healthy human being is fairly constant (36.5-37.5°C) despite fluctuating ambient temperatures, and this allows the biochemical processes to function unaffected by the outer conditions [5,15]. However, patients being anaesthetised in general, and injured patients in particular, face an inherent risk of hypothermia during procedures [5,8,11,19,20]. Hypothermia is technically recognised as a core temperature of less than 36.5°C, but a usual threshold for healthy subjects is < 35.0°C [8,15].

Hypothermia is further commonly divided into three groups depending on the core temperature (ranges for injured patients in brackets) [8]: mild 35-32°C (36-34°C); moderate 32-30°C (34-32°C); and severe < 30°C (< 32°C).

To prevent and combat hypothermia in burned patients in our unit we use Bair Hugger™, radiator ceilings, bed warmers (warm air), and Hotline^® ^or Fluido^®^- infusion heaters. However, we still encounter hypothermia occasionally in patients, both during and after procedures. Because of the warming technique used, the ambient room temperature is often negatively affecting the staff while working close to patients for long periods of time. We have also noticed that even though warm air is circulating around the patient from the bed warmers, patients with leaking wounds (and thus wet bandages) become even more hypothermic, probably as a result of strong convection effects cooling the patient.

This spurred us to investigate other ways of preventing and combating hypothermia in burn patients, while keeping the staff's working environment tolerable.

Many methods of heating have been reported in the quest for the optimal and most efficient technique [9,16,21-24], and two relatively new techniques appealed to us: the Allon™2001 Thermowrap (a temperature-regulating water mattress-fluid-convection), and Warmcloud (a temperature-regulating air mattress-air-convection).

The fluid-convection technique has been studied during thoracic and abdominal surgery and has been reported to increase (and maintain) body temperature more efficiently than traditional methods [12,22,23,25,26]. Extended details about the Allon™ 2001 Thermowrap are given by Nesher et al. 2001 [12]. There are fewer scientific reports about the KanMed Warmcloud [21,24].

We could find no reports of the use of either the fluid- or air-convection techniques for patients with burns, and so have investigated them in burned patients, and compared them with our ordinary heating technique.

The aim of this study was to investigate whether either the fluid-convection or the air-convection technique could prove to be more efficient in preventing and combating hypothermia in burn patients as compared to our conventional method.

## Patients and methods

The study was approved by the local ethics committee (Dnr 03-303) in accordance with the ethical principles that originated in the Declaration of Helsinki.

Inclusion criteria were patients with burns (> 20%TBSA-total body surface area), a core temperature of < 36.0°C, and oral and written informed consent. Consecutive patients with these criteria fulfilled were considered for inclusion on admission, or after procedures.

Patients were randomised to be treated with 'Allon'-Allon™2001 Thermowrap+ Hotline^® ^((Allon Thermowarp™, MTRE, Israel), (Hotline^® ^Blood and Fluid Warmer, Smiths Medical Sverige AB, Sollentuna, Sweden)), or 'Warmcloud'-Warm cloud + Hotline^® ^(KanMed Warmcloud, KanMed AB, Bromma, Sweden), or 'Conventional'-Bair Hugger^®^+ radiator ceiling + bed warmer + Hotline^® ^(Bair Hugger^®^, Medicvent AB, Umeå, Sweden).

All medical equipment including the Allon™2001 Thermowrap and KanMed Warmcloud was used according to the manufacturers' instructions.

### Procedure

The protocol was designed so that all patients were to be exposed to all 3 methods in a random fashion (according to a pre-study randomization scheme each patient could start with any one method and the other two followed randomly). Each treatment/measuring-cycle lasted for 6 hours, and should contain all three methods assessed for two hours each. Core temperature was measured every 15 minutes using an indwelling (bladder) thermistor.

To illustrate: a patient starts the study by being randomized to be rewarmed with the three techniques in the order of Conventional-Allon-Warmcloud. Thus the patient is subjected to firstly 2 hours of Bair Hugger^®^+ radiator ceiling + bed warmer + Hotline^® ^(Conventional heating), thereafter the Bair Hugger^® ^is removed, radiator ceiling and bed warmer turned off and the patient is put on the Allon™2001 Thermowrap mattress + Hotline^® ^for two hours (Allon heating), whereafter the Allon™2001 Thermowrap mattress is removed and the patient is put on the KanMed Warmcloud mattress + Hotline^® ^(Warmcloud heating) for two hours. Thus a 6-hour treatment/measuring-cycle is done with 2 hours assessment of each heating technique measuring core temperature every 15 minutes.

The planned 6-hour treatment/measuring-cycle was completed even if the patient reached a core temperature of > 36.0°C during the cycle. If a patient did not reach a core temperature of > 36.0°C within the 6-hour period, the same patient was included again, this time with a new randomisation protocol, and a subsequent 6-hour treatment/measuring-cycle.

### Statistical analysis

Paired *t*-tests were used to assess the significance of differences between the treatments within the patients. One-way analysis of variance (ANOVA) was used to assess the differences in temperature from the first to the last measurement among all treatments. Three-way ANOVA with the Tukey HSD *post hoc *test was used in the same manner, but included information about patients and treatment/measuring-cycles to control for potential confounding from ordering and also because not all patients received all treatments. A repeated measures ANOVA with information about all times and controlling for treatment, patients, and measuring-cycles for the same reason as above. Data are presented as mean (SD) and (range) if not otherwise stated. Probabilities of less than 0.05 were accepted as significant. The paired *t*-tests and one-way ANOVA were done with the help of StatView for Windows (version 5.0, SAS Institute Inc., Cary, NC, USA) and the three-way and repeated measures ANOVA were done using the Statistical Package for the Social Sciences (SPSS, version 17, SPSS Inc, Chicago, Ill, USA).

## Results

### General

A total of 10 patients that fulfilled the inclusion criteria of > 20% TBSA and a core temperature < 36.0°C were included in the study. The 10 patients (9 men, 1 woman) had a mean age of 48 years, mean body weight of 77 kg, and a mean total body surface area burned of 47%. The demographic data is shown in Table 1.

**Table 1:** Demography

Case No	Age (year)	Weight (kg)	TBSA-burn (%)	Full-thickness burn (%)	Deep dermal burn (%)	Superficial dermal burn (%)	No of treatment/measuring-cycles
**1**	32.0	80.0	87.0	45.0	42.0	0	1
**2**	58.0	76.3	56.0	41.5	4.5	0	4
**3**	26.0	72.0	59.0	39.0	17.0	3.0	2
**4**	42.0	90.0	21.5	2.0	15.5	4.0	2
**5**	41.0	87.0	20.0	0	3.5	16.5	1
**6**	78.0	78.4	20.5	2.0	18.5	0	1
**7**	78.0	70.0	32.5	20.5	11.0	1.0	1
**8**	20.0	66.0	56.0	27.5	28.5	0	2
**9**	66.0	74.1	27.0	24.5	1.5	1.0	3
**10**	36.0	80.0	86.0	79.5	6.5	0	1

Demography of patients including extent of burn and number of treatment/measuring-cycles. All where men except case 9.

The included patients gave rise to 18 treatment/measuring-cycles together with 21 'Allon', 23 'conventional', and 10 'Warmcloud' 2-hours periods. Of the 18 treatment/measuring-cycles 8 were completed with only the 'conventional' and 'Allon' methods however still in a randomized fashion. In these cases the 3 × 2-hour period with 15 minute intervals was followed, but the patient was exposed to only the two methods of heating. This was considered in the management of statistical data.

### Results of temperature changes

**Figure 1:**
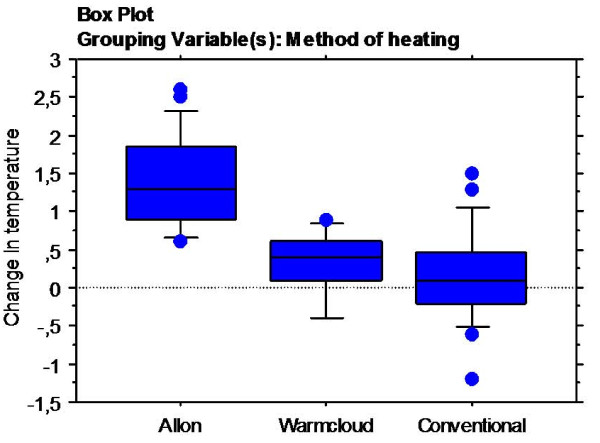
**Mean change in core temperature**. Box plot indicating the mean change in core temperature of the three methods of heating.

**Figure 2:**
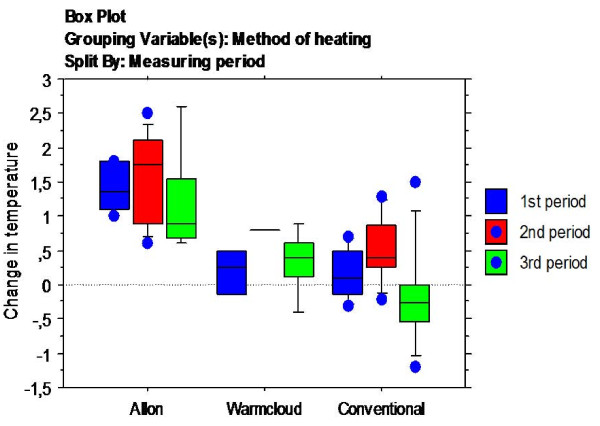
**Temperature changes split by 1^st^, 2^nd^, and 3^rd ^2-hour periods**. Box plot of temperature changes for the three methods of heating split by first, second, and third 2-hour periods during the 6-hour treatment/measuring-cycle.

Individual results are shown Table 2 and the complete data-set is found in Table 3: Appendix. 'Allon' increased the temperature in all 21 cycles and gave a mean change in temperature of 1.4 (0.6)°C (range 0.6-2.6) whereas 'conventional' showed a mean change in temperature of 0.2 (0.6)°C (range -1.2 to 1.5). 'Warmcloud' showed a mean temperature increase of 0.3 (0.4)°C (range -0.4 to 0.9) (Figure 1). The 2 hour-cycles of the 'conventional' heating technique either increased the patients' body temperature in 12/23 cycles, did not affect the temperature at all (1 cycle), or decreased the temperature in 10/23 cycles. Of the total 10 cycles the 'Warmcloud' increased the patients' body temperature in 8 (Table 2) and decreased the temperature in 2. There was significant differences (p < 0.0001) in temperature change in favour of 'Allon'. When split into first, second, and third 2-hour periods of the 6-hour treatment/measuring-cycle there were larger increases in core temperature with the 'Allon' than with the other two techniques for each period (p < 0.01, p < 0.05, and p < 0.05, respectively) (Figure 2).

**Table 2:** Change in core temperature per treatment/measuring-cycle

		Change in temperature from baseline (°C) during treatment/measuring-cycle of 2 hours		
		
Treatment/measuring-cycle	Case number	'Allon'	'conventional'	'Warmcloud'
1	1	1.3	0.5	0.5
2	2	1.2	0.4	0.4
3	2	1.8	0.2	0.4
4	2	1.1	-0.1	0.1
5	2	0.6	-0.3	0.9
6	3	1.8	-0.2	-0.4
7	3	1.8	-0.2/-0.4	
8	4	2.1	0.4/-0.2	
9	4	2.5	0.2/-0.1	
10	5	2.2	0/-0.5	
11	6	1.0/0.6	0.4	
12	7	1.7	0.6/-0.6	
13	8	1.4/0.9	1.0	
14	9	2.0	1.5	-0.4
15	8	0.9	-0.1	0.3
16	9	0.7	0.7	0.8
17	9	0.8	0.1	0.6
18	10	1.1/2.6	1.3	

**Mean (SD) temperature change (°C)**				
		1.43 (0.62)°C	0.16 (0.62)°C	0.32 (0.44)°C

Change in core temperature during each 2-hour cycle per treatment/measuring-cycle and technique. Note that in 10 treatment/measuring-cycles all 3 methods were completed and in 8 treatment/measuring-cycles just the 'conventional' and the 'Allon' methods were completed, however all 18 treatment/measuring-cycles were performed in a randomized fashion.

**Table 3:** Appendix

	Cycle 1		Cycle 2		Cycle 3		Cycle 4		Cycle 5		Cycle 6		Cycle 7		Cycle 8		Cycle 9	
	Pt 1		Pt 2		Pt 2		Pt 2		Pt 2		Pt 3		Pt 3		Pt 4		Pt 4	
Time	°C	Mod	°C	Mod	°C	Mod	°C	Mod	°C	Mod	°C	Mod	°C	Mod	°C	Mod	°C	Mod
**0'**	34,3		35,9		34,2		35,8		35,8		34,8		34,7		34,6		34,2	
**15'**	34,3	A	36	W	34,2	A	35,8	W	35,8	C	34,8	A	34,6	C	34,7	C	34,2	C
**30'**	34,6	A	35,9	W	34,5	A	35,8	W	35,6	C	34,9	A	34,7	C	34,8	C	34,2	C
**45'**	34,7	A	35,9	W	34,8	A	35,7	W	35,5	C	35,2	A	34,7	C	34,8	C	34,3	C
**60'**	34,6	A	36	W	35,2	A	35,7	W	35,4	C	35,4	A	34,7	C	35	C	34,3	C
**75'**	34,8	A	36,1	W	35,5	A	35,7	W	35,4	C	35,6	A	34,6	C	34,9	C	34,3	C
**90'**	35,3	A	36,2	W	35,7	A	35,7	W	35,4	C	36	A	34,7	C	34,9	C	34,4	C
**105'**	35,5	A	36,3	W	35,9	A	35,8	W	35,3	C	36,2	A	34,7	C	35	C	34,4	C
**120'**	35,6	A	36,3	W	36	A	35,9	W	35,5	C	36,6	A	34,5	C	35	C	34,4	C
**135'**	35,6	C	36,4	C	36,1	C	35,9	A	35,3	A	37	C	34	A	35,2	A	34,6	A
**150'**	36	C	36,5	C	36,1	C	36	A	35,3	A	36,8	C	34,1	A	35,5	A	34,9	A
**165'**	36	C	36,6	C	36	C	36,2	A	35,5	A	36,6	C	34,3	A	35,8	A	35,1	A
**180'**	36	C	36,6	C	36	C	36,5	A	35,5	A	36,9	C	34,6	A	36,1	A	35,5	A
**195'**	35,9	C	36,6	C	36,1	C	36,7	A	35,6	A	36,3	C	35	A	36,4	A	36	A
**210'**	36	C	36,6	C	36	C	36,7	A	35,7	A	36,7	C	35,3	A	36,6	A	36,3	A
**225'**	36	C	36,7	C	36,1	C	36,7	A	35,9	A	36,5	C	36	A	37	A	36,5	A
**240'**	36,1	C	36,7	C	36,2	C	37	A	36,1	A	36,4	C	36,3	A	37,1	A	36,9	A
**255'**	36,2	W	36,8	A	36,3	W	36,9	C	36,4	W	36,4	W	36,4	C	37	C	37,1	C
**270'**	36,3	W	36,9	A	36,3	W	37	C	36,6	W	36,3	W	36,3	C	36,8	C	37,1	C
**285'**	36,3	W	37	A	36,5	W	37	C	36,7	W	36,2	W	36,2	C	36,6	C	37,1	C
**300'**	36,4	W	37,2	A	36,6	W	37	C	36,8	W	36,1	W	36,1	C	36,3	C	36,9	C
**315'**	36,5	W	37,4	A	36,6	W	37	C	36,8	W	36	W	36	C	36,2	C	36,8	C
**330'**	36,5	W	37,4	A	36,6	W	36,9	C	36,9	W	35,8	W	35,9	C	36,1	C	36,8	C
**345'**	36,6	W	37,5	A	36,6	W	36,9	C	37	W	36	W	35,9	C	35,9	C	36,8	C
**360'**	36,6	W	37,9	A	36,6	W	36,9	C	37	W	36	W	35,9	C	35,9	C	36,8	C
																		
	**Cycle 10**		**Cycle 11**		**Cycle 12**		**Cycle 13**		**Cycle 14**		**Cycle 15**		**Cycle 16**		**Cycle 17**		**Cycle 18**	
	**Pt 5**		**Pt 6**		**Pt 7**		**Pt 8**		**Pt 9**		**Pt 8**		**Pt 9**		**Pt 9**		**Pt 10**	
**Time**	**°C**	**Mod**	**°C**	**Mod**	**°C**	**Mod**	**°C**	**Mod**	**°C**	**Mod**	**°C**	**Mod**	**°C**	**Mod**	**°C**	**Mod**	**°C**	**Mod**

**0'**	35		35,3		34,8		33,4		35,5		35,9		35,1		35,2		32,9	
**15'**	34,8	C	35,4	A	34,8	C	33,1	A	35,4	W	35,9	C	35,1	C	35,1	W	32,9	A
**30'**	34,8	C	35,5	A	34,7	C	33,1	A	35,3	W	35,8	C	35,3	C	35,1	W	32,8	A
**45'**	34,8	C	35,4	A	34,8	C	33,2	A	35,2	W	35,8	C	35,4	C	35,1	W	33	A
**60'**	34,8	C	35,7	A	34,9	C	33,5	A	35,3	W	35,7	C	35,6	C	35,2	W	33,2	A
**75'**	34,9	C	35,6	A	35	C	33,8	A	35,2	W	35,7	C	35,7	C	35,6	W	33,4	A
**90'**	34,9	C	35,7	A	35,2	C	34,2	A	35,2	W	35,8	C	35,7	C	35,6	W	33,6	A
**105'**	35	C	36,2	A	35,4	C	34,5	A	35,1	W	35,8	C	35,8	C	35,7	W	33,6	A
**120'**	35	C	36,3	A	35,4	C	34,8	A	35,1	W	35,8	C	35,8	C	35,8	W	34	A
**135'**	35,3	A	36,6	C	35,7	A	35,5	C	35,1	A	36	A	36	W	35,8	A	34,3	C
**150'**	35,3	A	36,7	C	36	A	35,6	C	35,3	A	36	A	36,1	W	35,9	A	34,5	C
**165'**	35,3	A	36,7	C	36,1	A	35,7	C	35,4	A	36,4	A	36,2	W	36	A	34,8	C
**180'**	35,8	A	36,6	C	36,3	A	35,7	C	35,9	A	36,5	A	36,2	W	36,1	A	34,7	C
**195'**	36	A	36,7	C	36,6	A	35,7	C	36,4	A	36,7	A	36,3	W	36,3	A	34,7	C
**210'**	36,7	A	36,6	C	36,7	A	35,8	C	36,7	A	37	A	36,4	W	36,4	A	34,7	C
**225'**	37	A	36,6	C	37	A	35,7	C	36,8	A	36,9	A	36,5	W	36,5	A	34,8	C
**240'**	37,2	A	36,7	C	37,1	A	35,8	C	37,1	A	36,7	A	36,6	W	36,6	A	35,3	C
**255'**	37,4	C	36,9	A	37,2	C	35,8	A	37,4	C	36,9	W	36,7	A	36,4	C	35,3	A
**270'**	37,1	C	37	A	37,2	C	36	A	37,4	C	36,9	W	36,7	A	36,4	C	35,3	A
**285'**	37	C	37,3	A	37	C	36	A	37,5	C	36,8	W	36,8	A	36,3	C	35,5	A
**300'**	36,9	C	37,4	A	37	C	36,1	A	37,9	C	36,9	W	36,9	A	36,4	C	35,8	A
**315'**	36,8	C	37,4	A	36,6	C	36,4	A	38,1	C	36,7	W	37	A	36,5	C	36,1	A
**330'**	36,8	C	37,4	A	36,6	C	36,4	A	38,2	C	36,8	W	37,1	A	36,6	C	37,8	A
**345'**	36,7	C	37,4	A	36,5	C	36,7	A	38,4	C	36,7	W	37,3	A	36,6	C	37,8	A
**360'**	36,7	C	37,3	A	36,5	C	36,7	A	38,6	C	37	W	37,3	A	36,7	C	37,9	A

The complete data-set of all treatment/measuring-cycles.

Pt-Patient, Mod-Modality, A-Allon (fluid-convection), W-WarmCloud (air-convection), C-Conventional

**Figure 3:**
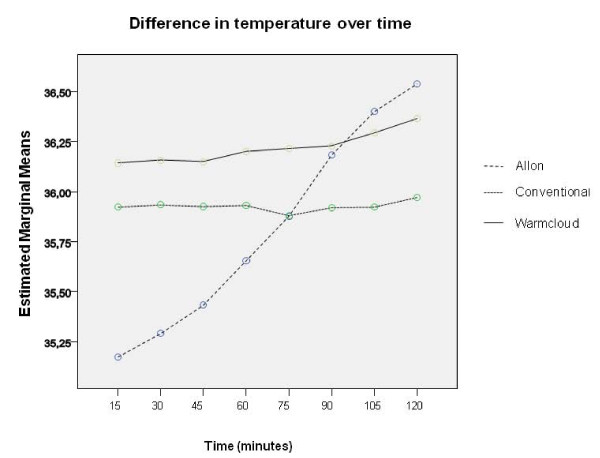
**Difference in temperature over time**. Difference in temperature over time adjusted for patient and treatment/measuring-cycle. Indicating specifically a significant interaction between heating technique 'Allon' and time. The lower starting temperature (statistically not significant) for 'Allon' was pure accidental as treating orders were randomized.

There was a significant difference between the methods of heating (p < 0.001) with a significant advantage for 'Allon' compared with both 'conventional' and 'Warmcloud' techniques (p < 0.001 in both cases). There was a significant interaction between the 'Allon' technique and time (Figure 3).

## Discussion

Transient hypothermia in burned patients is common. Because the burn itself includes damaged and severely disturbed thermoregulation and fluid regulation, burned patients are at risk of developing hypothermia, which is associated with increased mortality and morbidity. Several ways of reaching and maintaining a normal core temperature are available.

Burned patients also often become hypothermic during prehospital treatment and procedures, and it is difficult to rewarm patients, in part because their large open wounds leak fluid that increases heat loss by evaporation; leaking wounds also lead to wet bandages and so convective loss increases.

To be able to maintain a patient normothermic during resuscitation and treatment is important to avoid complications and for an adequate wound healing. A randomised controlled trial by Melling et al. showed that preoperative warming of patients significantly reduced the number of post operative infections [16].

The basic principle of increasing the ambient temperature is seldom sufficient, and the staff's work environment is also affected.

The fluid convection technique increased core temperature in all patients during all treatment/measuring-cycles, whereas the conventional method and the air-convection technique quite often either reduced the core temperature of the patients or increased it by only a small amount. The fluid convection technique was the only method that showed a significant correlation with time, that is, the longer the patient was left on the temperature regulating water-mattress, the more the core temperature increased (Figure 3).

The fact that 8 treatment/measuring-cycles consisted of just the conventional and the air-convection techniques has been considered in our analysis. However, even though the number of patients and treatment/measuring-cycles included is fairly small, a significant difference in increase in core temperature was shown among the different methods of heating in favour of the fluid-convection technique. This was true regardless of the order in which the patients were treated by each method (Figures 1 and 2). Still, a two-way experiment (with reverse-crossover) to confirm these findings would certainly be of considerable value. Nevertheless, these findings make us think that the results are relevant and may be generalizable to a larger group of patients and settings.

The superiority of the fluid-convection technique in increasing and maintaining body temperature better than the other heating techniques examined, based on intravenous fluid warmer and forced-air warmer in open heart and abdominal surgery (previously shown by Nesher et al. and Janicki et al.) [12,22] further strengthens our assumption that our findings are also generalizable to the burned patients.

Even though not studied in this paper, others have shown that the haemodynamic state of the patients is also improved when they are treated with fluid-convection technique as indicated by a higher cardiac index and lower systemic vascular resistance [12,22]. This is also of considerable value for burned patients but needs to be further studied.

The work environment also improved because the bulky and noisy bed-warmers (fans) could be omitted and the ambient temperature was improved (reduced to levels more comfortable to the staff) because the ceiling radiators could be turned off without the patients' core temperatures being reduced while warmed by the fluid-convection technique.

It is important to stress that the evaluation of the three warming techniques was done on patients that for some reason had developed mild hypothermia and the methods were evaluated on the point of ability to normalize the temperature in a short time perspective, i.e. 2 hours. An evaluation was not performed on the ability of the techniques to maintain normothermia. The latter effect may be less demanding for any technique.

It needs also to be pointed out that the conventional temperature controlling technique presented in this investigation has been developed and used for many years prior to this study and the burn treatment most commonly offered during the development of the technique has been the open exposure technique for burn care. This procedure is not used today and this is also an argument supporting the use of the fluid-convection technique. However, the changing and using another temperature regulating procedure also calls for an evaluation of how the temperature control systems may affect the whole burn care program and in the final end the wound healing process. The latter has not been evaluated in this study and needs also to be considered in the overall conclusions of the temperature regulating procedure used.

Nevertheless, it is obvious that the many adverse consequences of hypothermia in burn patients suggest that everything that can be done to prevent, or combat, low core temperature is important as to avoid increased morbidity and mortality.

## Conclusions

The fluid-convection technique (represented by the Allon™2001 Thermowrap) was more effective than both our conventional method and the air-convection technique (represented by the KanMed Warmcloud mattress) for the normalisation of accidental mild hypothermia in patients with significant burns. This technique also lead to an experienced improved work environment for the burn unit staff.

## Competing interests

The authors declare that they have no competing interests.

## Authors' contributions

All authors participated in the conceivement and design of the study; writing of drafts and manuscript; read and approved the final manuscript. BMK, FS, and FRMH carried out the practical clinical work. MF performed the statistical analysis.
